# Elongator is required for pattern recognition receptor and type I interferon signaling in macrophages

**DOI:** 10.1016/j.jbc.2025.110916

**Published:** 2025-11-05

**Authors:** Jamie Murphy, Marcin Baran, Corinna Grünke, Darya Haas, Gillian Barber, Andreas Pichlmair, Andrew G. Bowie

**Affiliations:** 1 School of Biochemistry and Immunology, Trinity Biomedical Sciences Institute, Trinity College Dublin, Dublin, Ireland; 2 Institute of Virology, School of Medicine, Technical University of Munich, Munich, Germany; 3 Institute of Virology, Helmholtz Center Munich, Munich, Germany; 4 German Centre for Infection Research (DZIF), Partner Site Munich, Germany

**Keywords:** innate immunity, interferon, interferon regulatory factor (IRF), lipopolysaccharide (LPS), macrophage, pattern recognition receptor (PRR), STAT transcription factor, Toll-like receptor 4 (TLR4), virus, elongator

## Abstract

Innate immune detection of pathogens by macrophages requires a rapid and large scale mobilization of cellular resources towards an appropriate immune response, mainly involving altered protein expression. Pathogen recognition is mediated by pattern recognition receptor (PRR) sensing of conserved microbial patterns, a prototypical example being recognition of lipopolysaccharide by Toll-like receptor 4. This leads to induction of pro-inflammatory cytokines, chemokines and type I interferons (IFNs) which together mobilize appropriate anti-pathogen responses. Type I IFNs signal to transcription of IFN-stimulated genes which encode numerous anti-viral proteins. Many studies have contributed to a detailed understanding of transcriptional regulation of mRNA during such responses, however much less is known about the contribution of protein complexes required for mRNA translation. Here we examine the role of the evolutionarily conserved Elongator complex in PRR signaling in macrophages. Elongator modifies tRNAs at U_34_ to facilitate more efficient wobble interactions between tRNAs and mRNA codons. In macrophages, deletion of *Elp3*, the catalytic subunit of Elongator, led to an impaired PRR-IFN I-IFN-stimulated gene signaling axis. The ELP3-dependent lipopolysaccharide-stimulated proteome was enriched with proteins involved in PRR activation of IRFs and in IFN signalling. Specifically, ELP3 was required for expression of key transcription factors regulating this axis, for TYK2-dependent type I IFN signaling and for IRF3 activation for some, but not all, PRRs. Importantly, ELP3 was also necessary for innate immune gene induction following virus infection. These data reveal specific roles for Elongator in PRR signaling and illustrate the underappreciated importance of translational regulation in optimal anti-pathogen innate immune responses.

Innate immune cells such as macrophages are tasked with mounting an appropriate response to pathogens to contain the pathogen and mobilise an innate and adaptive immune response. Critical to initiating this immune response is detection of pathogen-associated molecular patterns (PAMPs) by cellular pattern recognition receptors (PRRs), for example toll-like receptor 4 (TLR4) recognition of lipopolysaccharide (LPS) in gram negative bacterial cell walls, or sensing of viral nucleic acid by RNA sensors such as RIG-I-like receptors or by DNA sensors such as cGAS (1, 2, 3). Activation of PRR signaling pathways leads to induction of pro-inflammatory cytokines, chemokines and interferons (IFNs), an early indication of a mobilized immune response.

Clearly then, upon innate immune detection of pathogens, there is a need for a rapid and efficient synthesis of proteins such as cytokines and IFNs, and therefore gene expression needs to be both primed and tightly controlled for an anti-pathogen response. Transcriptional regulation of PRR responses has been well characterized, and the critical role of transcription factor families such as NFκB and IFN regulatory factors (IRFs) in PRR signalling well defined (4). However, comparatively less is known about the regulation of the next step in the signalling process of an innate immune response, namely the translation of mRNA to protein, and the role of protein complexes required for optimal mRNA translation. In this study, therefore, we have examined for the first time the role of the elongator complex, which increases the efficiency of translation of mRNA, in PRR signaling in macrophages.

Wobble interactions are one factor in controlling the efficiency of translation. There are 61 mRNA codons encoding amino acids. However, there are far fewer tRNA molecules for decoding of these codons, and as such, tRNAs possess the ability to interact with and decode multiple different mRNA codons. Standard interactions between an mRNA codon and the second and third nucleotide position of its cognate tRNA anticodon occur *via* Watson-Crick base pairing, involving adenine-uridine (A-U) or guanine-cytosine (G-C) pairs. ‘Wobble’ occurs at the first base (5′) of the tRNA anticodon (position 34), and third base (3′) of the mRNA codon. Here, the anticodon base can form a non-Watson Crick base pair with other nucleotides, such that U at position 34 (U_34_, wobble position) may pair with not only A (standard pairing) but also G (wobble pairing). However, wobble base pairs at U_34_ are unfavorable compared to canonical Watson-Crick interactions, due to poor base stacking and inefficient hydrogen bonding between unpaired nucleotides. To overcome the steric hindrance generated by unfavorable non-Watson-Crick wobble interactions, which would reduce translation efficiency, the U_34_ wobble position is chemically modified, in order to stabilize the anticodon stem loop of the tRNA and enhance base stacking (5, 6). The Elongator complex modifies tRNAs at U_34_ to facilitate wobble interactions when decoding mRNAs during translation.

Elongator catalyses the carboxymethylation (cm^5^) of wobble uridine U_34_ in tRNAs. Then, cm^5^U is further modified by other enzymes to 5-methoxycarbonylmethyl-uridine (mcm^5^U), 5-carbamoylmethyl-uridine (ncm^5^U) or 5-methoxycarbonymethyl-2-thiouridine (mcm^5^s^2^U) (7). These elongator-dependent modifications regulate tRNA binding in the A-site of ribosomes during elongation to facilitate optimal speed during polypeptide synthesis by stabilizing wobble interactions and preventing ribosome stalling (7). The elongator complex contains two copies of six subunits, ELP1-6, with ELP3 the catalytic subunit that binds the tRNA anticodon stem loop to modify the U_34_ nucleotide (8).

Elongator is highly conserved across the animal kingdom, with homologs of ELP3 being functionally present in higher organisms like humans, all the way back to Archaea that inhabit hydrothermal chimneys (9). Loss of an elongator subunit (ELP) in mammals is embryonic lethal (10) but conditional knock-out studies in differentiated cells and identification of ELP mutants has shed light on some elongator functions. For example, Elongator has been implicated in playing a vital role in neurodevelopment and neurodegeneration. Mutations in various Elongator subunits are associated with a profound spectrum of neurological conditions, for example *Elp1* mutations lead to development of Familial dysautonomia (11), while allelic variants of *Elp3* are associated with development of Amyotrophic lateral sclerosis (12). Aberrant activity of tRNA modifying-enzymes has the ability to induce the development of cancer, or translationally reprogram cells to a malignant phenotype (13). Thus, just as impaired levels of different Elongator subunits can lead to the development of neurological conditions, enhanced levels and activity of Elongator can lead to cancer and tumorigenicity.

However, the role of Elongator during an innate immune response in macrophages has not been investigated, even though these cells are rapid first responders to an infection, and it is unclear if, and how much, translation of immune-related genes after PRR activation require active elongator activity. Therefore, here we assessed the role of ELP3 in PRR responses in macrophages. Interestingly, certain but not all components of the PRR response were found to be ELP3-dependent, namely IRF3 activation by TLRs and RNA sensors, but not DNA sensors. Further, the type I IFN response was significantly impaired when ELP3 was absent, due to defective TYK2-dependent signalling.

## Results

### *Elp3*^*−/−*^ macrophages display an altered tRNA modification profile

**Figure 1:**
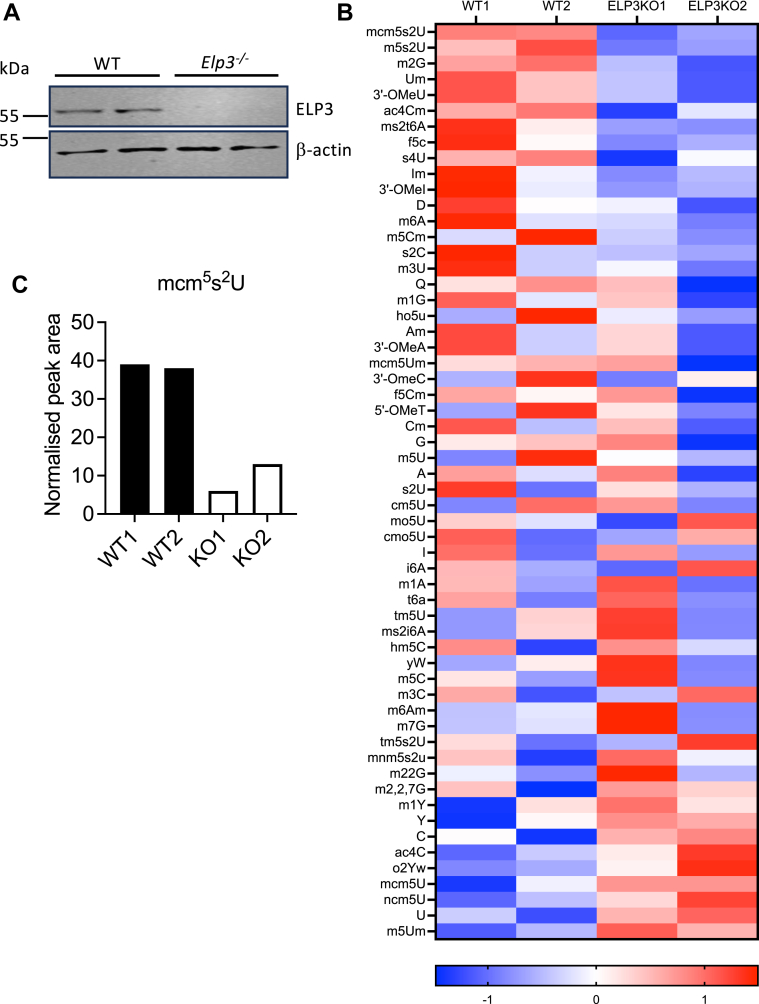
**Altered tRNA modification profile in *Elp3*^*−/−*^ macrophages**. *A*, cell lysates from WT and *Elp3*^*−/−*^ BMDMs were harvested and immunoblotted in duplicate for ELP3 and β-actin. Representative of three independent experiments. *B*, WT and *Elp3*^*−/−*^ BMDMs were seeded at 5 × 10^5^ cells/ml in 6-well plates in duplicate. RNA was isolated and tRNA modifications analysed by mass spectrometry. Heatmap shows normalized peak areas as Z-scores calculated for each row. *C*, the Elongator-dependent tRNA modification mcm^5^s^2^U is significantly impaired in *Elp3*^*−/−*^ cells. Data shows normalized peak area (LC-MS peak area normalized to quantity of injected tRNA), for individual duplicate samples. BMDM, bone marrow derived macrophage.

ELP3 is the catalytic subunit of Elongator that catalyses the modification of U_34_ in tRNAs. To study the function of elongator in innate immune responses we used CRISPR/Cas9 to generate *Elp3*^*−/−*^ macrophages using immortalized bone marrow derived macrophages (BMDMs) from Cas9-expressing mice. Western blotting of *Elp3*^*−/−*^ macrophages confirmed the loss of expression of ELP3 (Fig. 1*A*). Loss of any of the Elongator subunits is sufficient to disrupt the function of the complex (14), and analysis of tRNA chemical modifications in *Elp3*^*−/−*^ *versus* WT cells confirmed a defect in Elongator function, since the knock-out cells displayed a reduced level of mcm^5^s^2^U modification (Fig. 1, *B* and *C*). Reduction of mcm^5^s^2^U modifications was previously shown for ELP1 conditional knock-out mice (15) and for cells lacking ELP3 (16). The latter study showed a similar pattern of tRNA modifications in the absence of ELP3 as shown here, since the top 12 modifications that we found were reduced in *Elp3*^*−/−*^ macrophages (from mcm5s2U to D in Fig. 1*B*), were similarly reduced in mouse myeloid cells lacking ELP3 (16).

### Altered proteome in *Elp3*^*−/−*^ macrophages compared to WT cells

**Figure 2:**
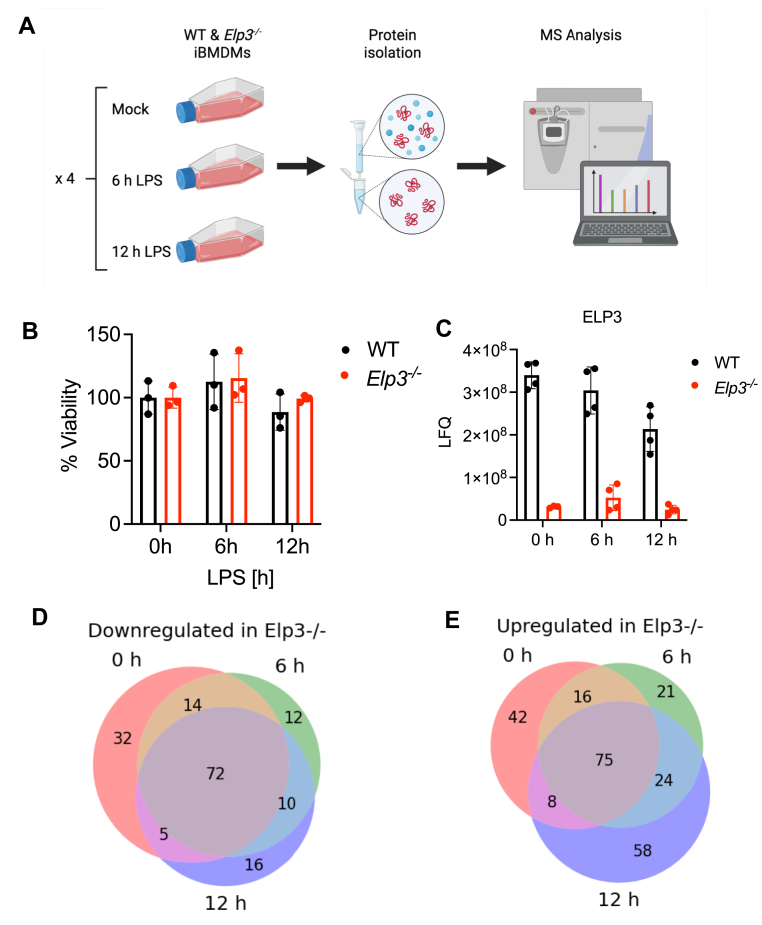
**Unbiased quantitative proteomic analysis of LPS-stimulated *Elp3*^*−/−*^ BMDMs**. *A*, schematic of workflow for unbiased quantitative proteomics analysis of iBMDMs. WT and *Elp3*^*−/−*^ BMDMs were seeded at 5 × 10^5^ cells/ml in 6-well plates in quadruplicate and then *left* untreated (mock) or stimulated with LPS (100 ng/ml) for 6 h or 12 h. Cell proteins were subsequently isolated and concentrations equalized before being processed by mass spectrometry for quantitative proteomic analysis. Figure created in BioRender. *B*, WT and Elp3^−/−^ BMDMs were seeded and treated as in (*A*). Cells viability was then analysed by MTT assay. Data are mean ± SD for triplicate samples, representative of two independent experiments. *C*, Label free quantification intensity values detected by mass spectrometry in WT and *Elp3*^*−/−*^ cells for peptides from ELP3. Data are mean ± SD for quadruplicate (or triplicate for WT 0 h sample) measurements. *D* and *E*, venn diagrams presenting distribution of both significant down down (*D*) and upregulated (*E*) proteins in *Elp3*^*−/−*^ cells compared to WT counterparts. Proteins were considered significantly up or downregulated if there was a log2 fold difference of 2 or greater in KO cells relative to their WT mock or LPS-stimulated counterpart. BMDM, bone marrow derived macrophage; iBMDM, Immortalised bone marrow–derived macrophage; LPS, lipopolysaccharide.

To study the potential role of Elongator in the macrophage innate immune response, we stimulated BMDMs with LPS to activate a TLR4-dependent signaling cascade. We utilized an untargeted LC-MS/MS approach using data-dependent acquisition to quantitatively profile the proteome of *Elp3*^*−/−*^ BMDMs both basally and following LPS stimulation, compared to WT cells. Cells were left untreated or stimulated with LPS for 6 h or 12 h in quadruplicate, and then harvested for proteomic analysis by mass spectrometry (Fig. 2*A*). In parallel, a thiazolyl blue tetrazolium bromide (MTT) assay was used to monitor cell metabolic activity, as an indicator of cell viability, which showed that viability of *Elp3*^*−/−*^ cells was hardly affected relative to their mock and LPS-stimulated WT counterparts (Fig. 2*B*). This suggested that any potential effects on protein expression levels in the absence of ELP3 would not result from elevated levels of cell death. Table S1 shows the label free quantification (LFQ) intensity of peptides detected by mass spectrometry assigned to 6533 mouse proteins. Figure 2*C* shows LFQ intensity for ELP3 detected in unstimulated WT and *Elp3*^*−/−*^ BMDMs, confirming minimal detection of ELP3 in *Elp3*^*−/−*^ BMDMs.

Proteomic analysis revealed 405 proteins that were differentially expressed in *Elp3*^*−/−*^ cells as compared to WT cells (Fig. 2, *D* and *E*). 161 proteins were downregulated in the absence of ELP3 (Fig. 2*D*), while 244 proteins were found to be increased in *Elp3*^*−/−*^ cells compared to WT cells (Fig. 2*E*). Therefore, disruption of Elongator has a significant effect on both the constitutive and the LPS-stimulated proteome in macrophages.

### The ELP3-dependent LPS-stimulated proteome is enriched for proteins involved in IFN signalling

A Gene Ontology (GO) enrichment analysis was performed on significantly upregulated or downregulated proteins identified in each comparison: *Elp3*^*−/−*^ *versus* WT at 0 h (mock), 6 h LPS stimulation, and 12 h LPS stimulation. No terms met the significance threshold in the upregulated protein sets; therefore, only results from the downregulated sets are shown as a heatmap. The data is presented in Figure S1 showing the top four terms are *innate immune response*, *cellular response to LPS*, *defense response to virus* and *negative regulation of viral genome replication*.

**Figure 3:**
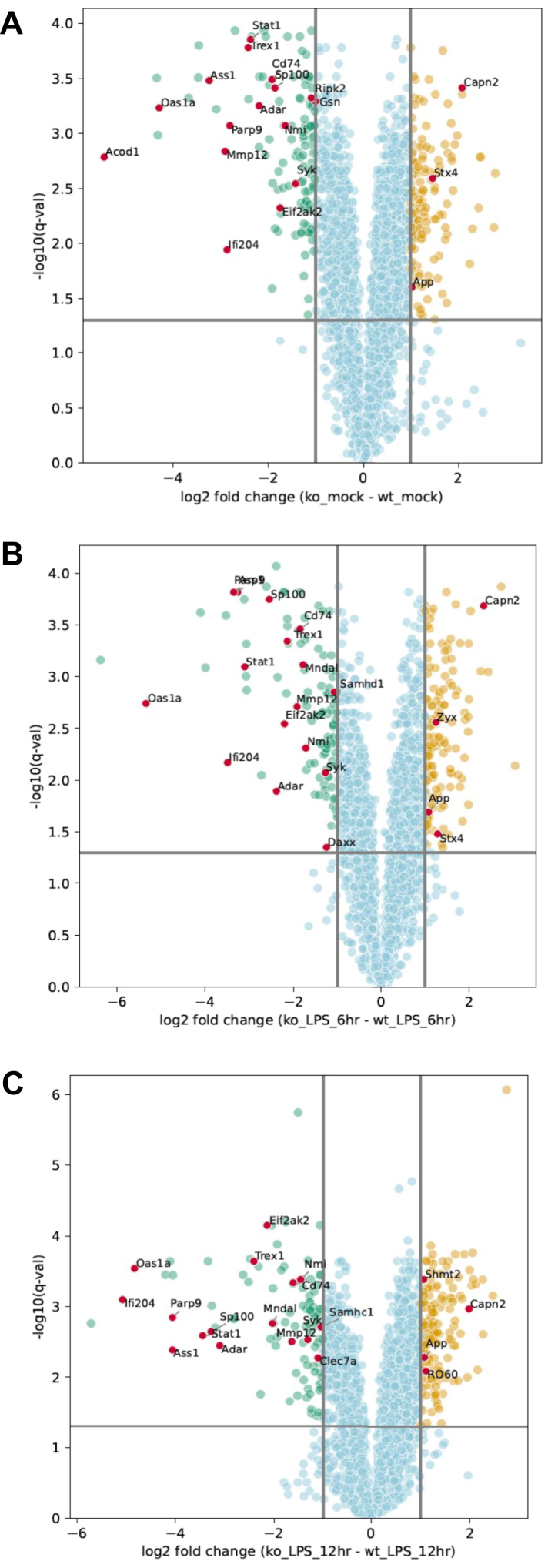
**Differential protein expression in *Elp3*^*−/−*^ BMDMs relative to WT cells**. Volcano plots showing differentially expressed proteins in unstimulated (*A*), 6 h LPS-treated (*B*) or 12 h LPS-treated (*C*) *Elp3*^*−/−*^ lysates *versus* lysates from WT cells. To capture a broad range of biological processes potentially influenced by loss of ELP3 an enrichment cut-off of a –log10 (*p*-value) of 1.3 was considered significant. Proteins associated with IFN signalling by gene ontology biological process terms are labelled as *red dots*. IFN, interferon; LPS, lipopolysaccharide.

Figure 3, *A*–*C* shows the distribution and identity of differentially expressed proteins in unstimulated and LPS-stimulated cells as represented by volcano plots. Upon examination of proteins downregulated in *Elp3*^*−/−*^ cells relative to WT cells, consistent with the GO enrichment analysis, we noticed an enrichment of proteins associated with IFN signalling, based on GO biological process terms (labelled as red dots in Fig. 3, *A*–*C*). Specifically, many of the proteins are expressed from IFN-stimulated genes (ISGs) known to be upregulated by type I IFN. The type I IFN system in macrophages constitutes a biologically-relevant immune response to signalling by many PAMPs including LPS. Thus, the fact that both basal and LPS-stimulated proteins related to IFN signalling were ELP3-dependent warranted further investigation.

### Impaired LPS-IFN I signalling axis in *Elp3*^*−/−*^ BMDMs

**Figure 4:**
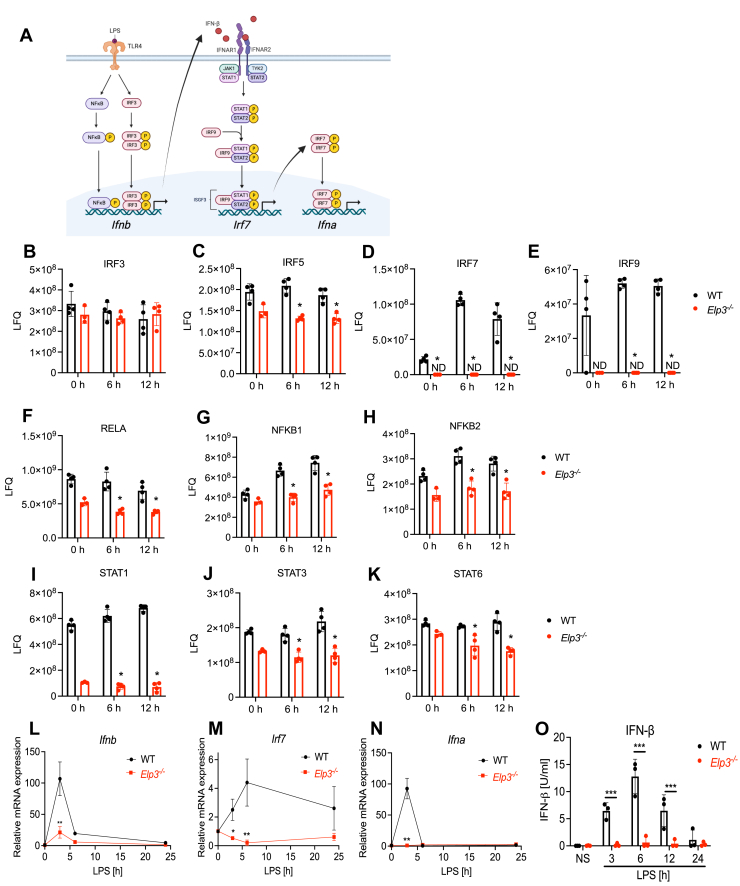
**ImpairedLPS-IFNI signalling axisin*****Elp3***^***−/−***^**BMDMs.***A*, schematic of LPS- IFN-I signalling axis. Figure created in BioRender (*B*–*K*) Label free quantification intensity values detected by mass spectrometry in WT and *Elp3*^*−/−*^ cells stimulated for 0, 6 or 12 h with LPS for peptides from transcription factor proteins IRF3 (*B*), IRF5 (*C*), IRF7 (*D*), IRF9 (*E*), RELA (*F*), NFKB1 (*G*), NFKB2 (*H*), STAT1 (*I*), STAT3 (*J*) and STAT6 (*K*). ND, not detected. Data are mean ± SD for quadruplicate (or triplicate for WT 0 h sample) measurements. ∗*p* < 0.05 compared to WT, based on Mann-Whitney test. *L*–*N*, WT and *Elp3*^*−/−*^ cells were stimulated with LPS (100 ng/ml) for 3, 6 and 24 h. *Ifnb* (*L*), *Irf7* (*M*) and *Ifna* (*N*) mRNA were assayed by qRT-PCR, expressed relative to the untreated WT control and normalized to the housekeeping gene β-actin. Data are mean ± SD of three independent experiments. Data significance was tested with a 2-way ANOVA using Šídák's multiple comparisons test. ∗*p* < 0.05 and ∗∗*p* < 0.0001 compared to WT. *O*, cells were stimulated with LPS for the indicated times and supernatants assayed by ELISA for IFN-β. Data are mean ± SD for three independent experiments, each performed on three WT or *Elp3*^*−/−*^ clones in triplicate. ∗∗∗*p* < 0.001. LPS, lipopolysaccharide.

A schematic of the LPS-stimulated type I IFN signalling cascade leading to induction of ISGs such as *Irf7* is shown in Figure 4*A*. Three key transcription factor families involved in this cascade are the IRF, NFκB and STAT families. Thus, any dependency of these transcription factors on ELP3 for their expression could explain the requirement of ELP3 for ISGs. We therefore interrogated the proteomic data for these three transcription factor families in WT compared to *Elp3*^*−/−*^ cells.

For IRF family members, LFQ intensity for IRF3, which is critical for LPS-stimulated IFNβ induction, was very similar in WT and KO cells (Fig. 4*B*) while in comparison IRF5 expression was lower in KO cells compared to WT cells (Fig. 4*C*). Interestingly, expression of IRF9, required for IFNβ-stimulated ISG expression, and IRF7, required for *Ifna* induction, were both increased after LPS stimulation in WT cells but virtually absent in KO cells (Fig. 4, *D* and *E*). For the NFκB family, whose members contribute to the initial transcriptional response to LPS, including *Ifnb* induction, each of RELA (p65), NFKB1 (p50) and NFKB2 (p52) were reduced in both unstimulated and stimulated cells (Fig. 4, *F*–*H*). Notably, STAT1, a major transcription factor controlling much of type I IFN signaling for ISG induction, was greatly reduced in both unstimulated and stimulated *Elp3*^*−/−*^ cells compared to WT cells (Fig. 4*I*). In contrast, there was a more minor reduction of other detectable STAT family members STAT3 and STAT6 in *Elp3*^*−/−*^ cells (Figure 4, *J*, *K*), while STAT2 expression was low with only a few peptides detected in WT cells and none in KO cells (Table S1). Hence, expression of multiple transcription factors key for the LPS-IFN I signaling axis is impaired in *Elp3*^*−/−*^ cells.

### LPS-mediated induction of type I IFNs and *Irf7* is impaired in *Elp3*^*−/−*^ macrophages

As several key transcription factors in the TLR4–type I IFN signaling pathway (NFκB family proteins, IRF7, IRF9, STAT1) were reduced in *Elp3*^*−/−*^ cells, we next assessed the impact on *Ifnb*, *Irf7*, and *Ifna* mRNA induction after LPS stimulation (Fig. 4*A*). Notably, in all three cases LPS-induced mRNA induction was significantly impaired in *Elp3*^*−/−*^ cells compared to WT cells (Fig. 4, *L*–*N*). Specifically, LPS-stimulated *Ifnb* induction, which is IRF3- and NFκB-dependent (Fig. 4*A*) was significantly reduced in *Elp3*^*−/−*^ cells, as was secretion of IFN-β protein (Fig. 4*O*). Thus, impaired IFN-β secretion might account for the impairment in both LPS-induced *Irf7* and *Ifna* mRNA (Fig. 4, *M* and *N*) since these gene induction events are dependent on IFNβ signaling (Fig. 4*A*). Also, or alternatively, given basal STAT1 expression was ELP3-dependent (Fig. 4*I*), as was LPS-induced IRF9 (Fig. 4*E*), defects in type I IFN signaling in the absence of ELP3 could also account for the impaired *Irf7* and subsequent *Ifna* induction (Fig. 4*E*). Therefore, we next examined the effect of elongator deficiency directly on type I IFN signalling.

### ELP3 is required for IFN-β-stimulated STAT1 activation and ISG induction

**Figure 5:**
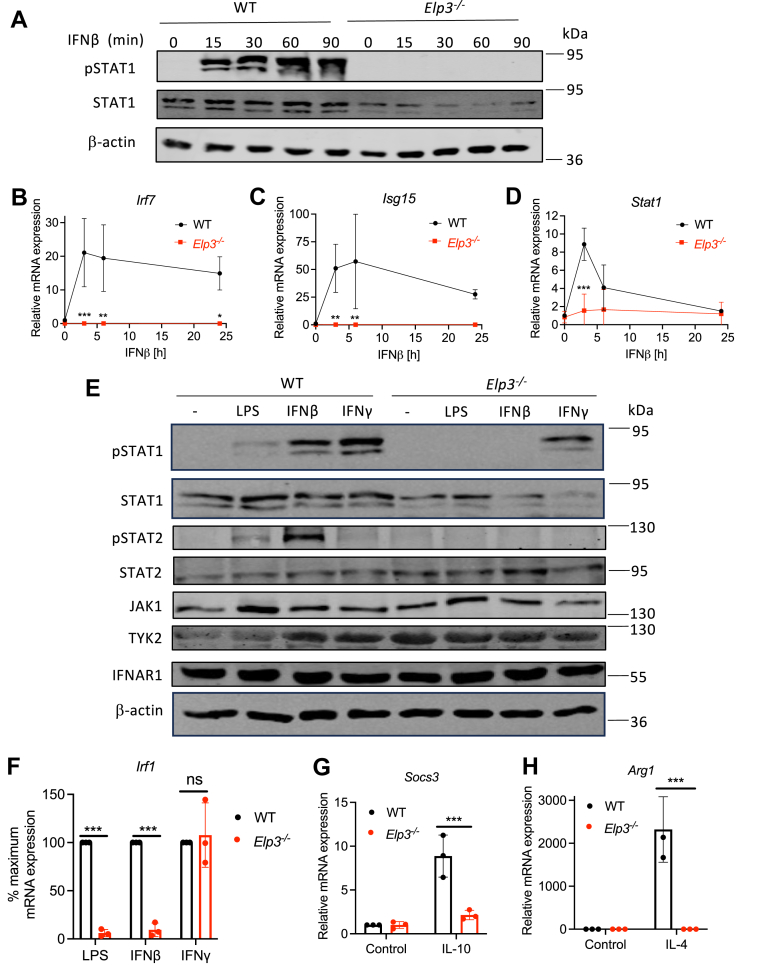
**ELP3 is required for IFN I and TYK2-dependentsignaling.***A*, WT and *Elp3*^*−/−*^ BMDMs were stimulated with 1000 U/ml IFNβ for the indicated times. Cells were harvested and immunoblotted for phosphorylated STAT1, STAT1 and β-actin. Representative of three independent experiments. *B*–*D*, WT and Elp3^−/−^ BMDMs were stimulated with 1000 U/ml IFNβ for the indicated times. RNA was isolated and mRNA expression of *Irf7* (*B*), *Isg15* (*C*), and *Stat1* (*D*) were assayed by qRT-PCR, expressed relative to the untreated WT control and normalized to β-actin. *E*, WT and *Elp3*^*−/−*^ iBMDMs were stimulated with either LPS (100 ng/ml), IFNβ (1000 U/ml) or IFNγ (25 ng/ml) for 90 min. Cells were harvested and expression of the indicated proteins was assessed by immunoblot. Representative of 3 independent experiments. *F*, WT and *Elp3*^*−/−*^ iBMDMs were stimulated with either LPS (100 ng/ml), IFNβ (1000 U/ml) or IFNγ (25 ng/ml) for 3 h. *Irf1* gene expression was assessed by qRT-PCR. mRNA levels are represented relative to β-actin, with each WT sample set to 100% and *Elp3*^*−/−*^ mRNA levels expressed as a percentage of their WT stimulated counterpart. *G*, WT and *Elp3*^*−/−*^ iBMDMs were stimulated with IL-10 (10 ng/ml) for 3 h. *Socs3* mRNA was assayed by qRT-PCR, expressed relative to the untreated WT control and normalized to the housekeeping gene β-actin. *H*, WT and *Elp3*^*−/−*^ iBMDMs were stimulated with IL-4 (10 ng/ml) for 3 h. *Arg1* mRNA was assayed by qRT-PCR, expressed relative to the untreated WT control and normalized to the housekeeping gene β-actin. For (*B*–*D* and *F*–*H*), data are mean ± SD of 3 independent experiments, each performed in triplicate. Data significance was tested with a 2-way ANOVA using Šídák's multiple comparisons test. ∗*p* < 0.05 and ∗∗*p* < 0.01 and ∗∗∗*p* < 0.001 compared to WT. iBMDM, immortalised bone marrow–derived macrophage; LPS, lipopolysaccharide.

To assess the effect of ELP3-deficiency on type I IFN signalling, we directly stimulated WT and *Elp3*^*−/−*^ macrophages with IFNβ and measured phosphorylation of STAT1 and induction of ISGs by immunblot assay. IFNβ signaling was inhibited in the absence of Elongator, since phospho-STAT1, easily observable in WT cells, was completely undetectable in stimulated *Elp3*^*−/−*^ cells (Fig. 5*A*). Figure 5*A* also shows reduced total STAT1 protein in *Elp3*^*−/−*^ cells, consistent with the proteomic analysis (Fig. 4*I*). Induction of mRNAs of ISGs *Irf7*, *Isg15* and *Stat1* measured in response to IFN-β stimulation were also impaired in *Elp3*^*−/−*^ cells (Fig. 5, *B*–*D*).

Given these results showing impaired type I IFN signaling and reduced induction of ISG mRNA, we wondered whether Elongator might have a broader role in ISG protein expression in general — especially since induced expression of ISG-encoded IRF7 and IRF9 was severely compromised in *Elp3*^*−/−*^ cells (Fig. 4, *D* and *E*), as were many other known ISGs (Fig. 3). We considered a list of over 100 ISGs identified as common to mouse and human cells (17), and found that 83 of these were detectable in the proteome data. Many, but not all, of these ISGs were reduced in expression in *Elp3*^*−/−*^ compared to WT macrophages. Figure S2, *A*–*L* shows results for 12 representative ISG-encoded proteins, some of which were inducible by LPS (*e*.*g*.*,* IFI204 and IFIT3) while others were not (*e*.*g*.*,* USP25 and ADAR). Interestingly, basal expression of many ISGs was also reduced in the absence of ELP3. This could be due to tonic type I IFN signaling being required for expression of a given ISG (since type I IFN signaling was impaired in *Elp3*^*−/−*^ cells), due to their expression being dependent on another ELP3-dependent protein, or basal expression of ISG proteins could be ELP3-dependent due to the mRNAs for such proteins having a high content of elongator-dependent codons (EDCs). To assess which ISGs would be predicted to be directly elongator-dependent due to a high content of codons requiring elongator-dependent modification for optimal protein expression, we assessed predicted elongator codon-dependency. Previous studies showed that codons AAA, CAA, or GAA caused more translation elongation defects in the absence of the mcm^5^s^2^U chemical modification of U_34_ in tRNAs (18, 19) (a modification confirmed to be reduced in the *Elp3*^*−/−*^ cells here (Fig. 1*C*)). Thus, to assess EDCs in a given gene, we calculated the percentage of AAA, CAA, and GAA codons in the coding region of the mRNA. Of the 12 representative ISGs shown, Figure S2*M* shows that seven displayed a high percentage of EDCs (arbitrarily defined as more than 10% since previous publications indicate significant ELP-dependency for such genes (16, 20, 21)), while none of the transcription factors described in Figure 4 did. However, some ISGs with a low EDC content were also reduced in the absence of ELP3 (Fig. S2, *I*, *J*, *L*), so there was no direct correlation between the percentage of EDCs in an ISG and their protein expression in the absence of ELP3.

### Elongator is required for IFN I and TYK2-dependent signaling

We next examined in more detail why direct type I IFN signaling was ELP3-dependent. Figure 4*A* showed that IFNβ-stimulated phosphorylation of STAT1 was significantly impaired in *Elp3*^*−/−*^ cells. While reduced STAT1 expression in Elp3^−/−^ cells (Figs. 4*I* and 5*A*) could underlie some defects, we observed that STAT1 phosphorylation in response to LPS and IFNβ, but not IFNγ, was selectively impaired (Fig. 5*E*). Concurrently, LPS- and IFNβ-, but not IFNγ-, stimulated transcription were impaired in *Elp3*^*−/−*^ cells as measured by induction of *Irf1* mRNA in response to all three stimuli (Fig. 5*F*). Thus, in principle there is sufficient STAT1 protein remaining in *Elp3*^*−/−*^ cells to allow productive STAT1-dependent signalling for IFNγ at least.

Further evidence for a defect in type I IFN signaling distinct from reduced STAT1 protein levels was obtained when STAT2 activation was measured, since IFNβ- (and LPS-) stimulated phospho-STAT2 was inhibited in *Elp3*^*−/−*^ cells compared to WT cells (Fig. 5*E*), even though levels of STAT2 protein were not reduced in the absence of ELP3 (Fig. 5*E*).

Impaired IFNβ signaling was not due to reduced expression of key signaling components required for STAT phosphorylation—IFNAR1, JAK1, or TYK2—as none of these proteins were decreased in lysates from *Elp3*^*−/−*^ cells (Fig. 5*E*). Both IFNβ and IFNγ signaling pathways utilize JAK1, but a key difference is that TYK2 is required only for IFNβ signaling. Although TYK2 protein levels were similar in WT and *Elp3*^*−/−*^ cells, we hypothesized that Elongator may be necessary for TYK2 activation. To test this, we examined other TYK2-dependent pathways by stimulating cells with IL-4 and IL-10 and measuring induction of Socs3 and Arg1 mRNA, respectively. Both IL-4- and IL-10-induced gene expression were impaired in *Elp3*^*−/−*^ cells compared to WT (Fig. 5, *G* and *H*), indicating that Elongator is essential for TYK2-dependent signaling pathways in addition to type I IFN signaling.

### Distinct PRR response pathways require elongator for signalling *via* IRF3

**Figure 6:**
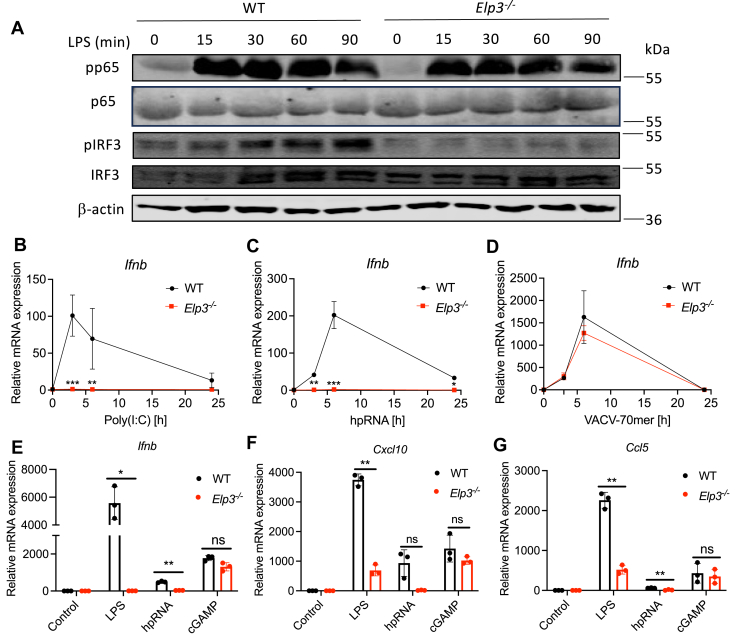
**Elp3 is required for TLR- andRIG-I-but notSTING-mediatedsignalling.***A,* WT and Elp3^−/−^ iBMDMs were stimulated with LPS (100 ng/ml) for the indicated times. Cells were harvested, and total and phosphorylated IRF3 and p65 were assessed by immunoblot. Representative of 3 independent experiments. (*B*–*D*) WT and Elp3^−/−^ iBMDMs were transfected with either 10 μg/ml Poly(I:C) (*B*), 200 ng/ml 3p-hpRNA (*C*) or 2.5 μg/ml VACV-70mer (*D*) for the indicated times. mRNA expression of *Ifnb* was assayed by qRT-PCR, expressed relative to the untreated WT control and normalized to the housekeeping gene β-actin. Data are mean ± SD of 3 independent experiments. Data significance was tested with a 2-way ANOVA using Šídák's multiple comparisons test. ∗*p* < 0.05 and ∗∗*p* < 0.01 and ∗∗∗*p* < 0.001 compared to WT. *E* and *G*, WT and Elp3^−/−^ iBMDMs were stimulated with either LPS (100 ng/ml) or transfected with either 3p-hpRNA (200 ng/ml) or 2′-3′ cGAMP (5 μg/ml) for 3 h mRNA expression of *Ifnb* (*E*), *Cxcl10* (*F*) or *Ccl5* (*G*) was assayed by qRT-PCR, expressed relative to the untreated WT control and normalized to the housekeeping gene β-actin. Data are mean ± SD of 3 independent experiments, each performed in triplicate. ∗*p* < 0.05 and ∗∗*p* < 0.01 compared to WT, based on paired *t* test. LPS, lipopolysaccharide.

Having shown that direct type I IFN signaling was ELP3-dependent, we next addressed the earlier issue of why LPS-induced *Ifnb* mRNA induction was ELP3-dependent (Fig. 4*L*). We tested whether reduced expression of NFκB family members (Fig. 4, *F*–*H*) fully explains the impaired LPS-induced *Ifnb* induction or if other mechanisms contribute to this. Therefore, we measured activation of NFκB and IRF3 by immunoblot assay, both of which are required for LPS-stimulated *Ifnb* induction. This showed that NFκB activation after LPS stimulation was normal in *Elp3*^*−/−*^ cells, as measured by p65 (RelA) phosphorylation (Fig. 6*A*), even though NFκB proteins were reduced in *Elp3*^*−/−*^ cells (Fig. 4, *F*–*H*). We tested two LPS-inducible NFκB-dependent genes, *Il6* and *Tnf*, and found that although there was some reduction of mRNA in *Elp3*^*−/−*^ cells, unlike the case for *Ifnb*, this was did not translate to an effect on IL-6 or TNF protein in ELISAs (data not shown). Interestingly, in contrast to NFκB, even though IRF3 protein expression was unaffected by the absence of ELP3 (Figs. 4*B* and 6*A*), LPS-stimulated IRF3 phosphorylation was apparent in WT cells but undetectable in *Elp3*^*−/−*^ cells (Fig. 6*A*). Thus, impaired IRF3 activation likely accounted for the lack of LPS-stimulated *Ifnb* mRNA induction. This data suggests that ELP3 is required for activation of IRF3 downstream of TLR4 signaling.

Importantly, in a separate ELP3 loss of function system, involving reduction of *ELP3* expression by shRNA in human monocytic THP-1 cells (Fig. S3*A*), LPS-stimulated *IFNB* induction was significantly impaired when *ELP3* expression was reduced (Fig. S3*B*), as was another IRF3-dependent gene, *CXCL10* (Fig. S3*C*). In contrast there was no significant reduction of the NFκB-dependent gene *TNF* (Fig. S3*D*).

We therefore wondered whether other PRR sensing pathways known to activate IRF3 for *Ifnb* induction were also ELP3-dependent. Poly(I:C) is a synthetic dsRNA analog that mimics an RNA virus infection. Poly(I:C) transfection leads to sensing *via* the cytosolic RNA sensor MDA5 (22). We transfected WT and *Elp3*^*−/−*^ cells with poly(I:C) for 3, 6 and 24 h and measured *Ifnb* mRNA induction. Gene induction of *Ifnb* was completely ablated in cells lacking ELP3 (Fig. 6*B*). This was also the case when we stimulated cells with transfected 3p-hpRNA, a specific ligand for the cytosolic RNA sensor RIG-I (22) (Fig. 6*C*). Interestingly, not all IRF3-dependent nucleic acid sensing pathways were affected by absence of Elongator, since *Ifnb* mRNA elicited by transfection of dsDNA, which is sensed by cGAS and other intracellular DNA sensors acting through the adaptor STING, was normal in *Elp3*^*−/−*^ BMDMs (Fig. 6*D*). To further clarify the differential role of Elongator in IRF3-dependent PRR sensing pathways we directly compared the ability of LPS, 3p-hpRNA and cGAMP (which directly activates STING) to induce three IRF3-dependent cytokines, namely *Ifnb*, *Cxcl10* and *Ccl5*, which showed that LPS- and 3p-hpRNA-, but not cGAMP-stimulated mRNA induction was ELP3-dependent (Fig. 6, *E*–*G*).

Thus, the data shows that ELP3 is required for signalling and downstream gene induction of TLR4 and RLR pathways, whilst ELP3 was not necessary for STING-mediated DNA sensing responses.

### ELP3 is necessary for innate immune gene induction following RNA virus infection

**Figure 7:**
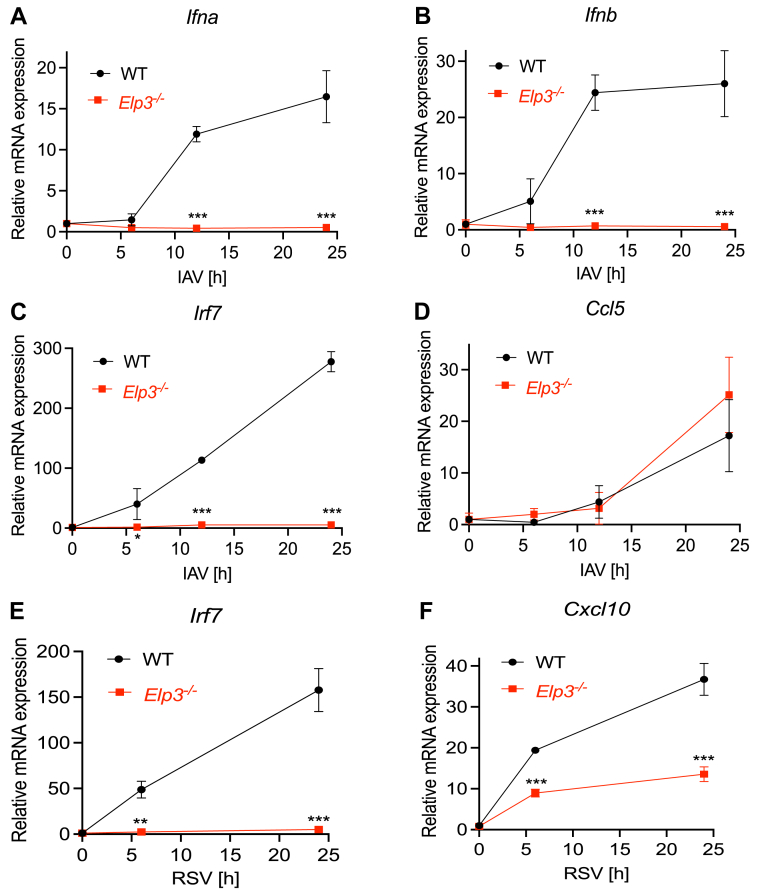
**Elp3 regulates innate immune responses to RNA viruses in macrophages**. *A and D*, WT and Elp3^−/−^ iBMDMs were seeded at 5 × 10^5^ cells/ml and infected with PR8 strain of IAV (MOI of 5) for the indicated times. mRNA expression of *Ifna* (*A*), *Ifnb* (B), *Irf7* (*C*) and *Ccl5* (*D*) was assayed by qRT-PCR, expressed relative to the untreated WT control and normalized to the housekeeping gene β-actin. *E and F*, Cells were infected with RSV (MOI of 5) for the indicated times. mRNA expression of *Irf7* (*E*) and *Cxcl10* (*F*) was assayed by qRT-PCR, expressed relative to the untreated WT control and normalized to the housekeeping gene β-actin. Data are mean ± SD of 3 independent experiments, each performed in triplicate. Data significance was tested with a 2-way ANOVA using Šídák's multiple comparisons test. ∗*p* < 0.01 and ∗∗*p* < 0.001 and ∗∗∗*p* < 0.0001 compared to WT. IAV, influenza A virus.

Since we found that ELP3 was required for IRF3-dependent signaling to cytokine induction for RNA PAMP sensing, we next considered a role for Elongator in innate immune gene induction following RNA virus infection of macrophages. We infected WT and *Elp3*^*−/−*^ macrophages with the mouse-adapted PR8 H1N1 strain of influenza A virus (IAV). Upon PR8 infection, *Elp3*^*−/−*^ cell-mediated type I IFN expression (both *Ifna* and *Ifnb*) was completely ablated relative to WT cells (Fig. 7, *A* and *B*), as was *Irf7* mRNA induction (Fig. 7*C*). However *Elp3*^*−/−*^ cells upregulated *Ccl5* to levels comparable with WT cells following PR8 viral infection (Fig. 7*D*), so not all host innate gene induction responses to IAV were Elongator-dependent. We also examined host innate gene induction responses to another RNA virus, RSV, sensed by both RLRs and extracellular TLR4 (23). RSV-mediated induction of type I IFNs was undetectable following infection of either WT and *Elp3*^*−/−*^ cells (data not shown), likely due to immune evasion strategies employed by the virus. However, other genes such as *Irf7* and *Cxcl10* were inducible by RSV infection, and both of these gene inductions were impaired in cells lacking ELP3 (Fig. 7, *E* and *F*). These data indicate that ELP3 is required for induction of innate immune genes that are important in coordinating antiviral immunity to RNA viruses IAV and RSV.

Overall, these data indicate a two-step requirement for ELP3 in PRR-mediated type I IFN responses. Firstly, ELP3 is necessary for TLR4-and RLR-mediated IRF3 activation and initial IFNβ gene induction. Secondly, ELP3 coordinates type I IFN signalling, STAT1 activation and gene induction responses possibly *via* a requirement for TYK2 activation.

## Discussion

Elongator modifies tRNAs at U_34_ through the catalytic ELP3 subunit to facilitate wobble interactions when decoding mRNAs during translation. Many studies in various model organisms and in mammalian cells have shown that the evolutionarily conserved role of Elongator is to chemically modify U34 and that absence of an Elongator complex subunit results in the loss of ncm5U-, mcm5U- and mcm5s2U-modified wobble nucleosides in tRNA (14, 24, 25). Ribosomal profiling shows that lack of the mcm5s2U wobble modification in tRNA increases pausing of ribosomes when the AAA, CAA or GAA codon is in the ribosomal A-site (18, 19), while the presence of this modification reduces ribosomal frameshifting (26). Since innate immune responses to PAMPs and viruses require rapid and efficient synthesis of proteins such as cytokines and IFNs, it would be expected that the process of protein translation would need to be operating at a highly efficient level. Despite this, the regulation of translation post-PRR stimulation has not been examined in as much detail compared to transcription. Hence, here we examined the role of the Elongator complex post-PRR stimulation of macrophages by examining the proteome and gene induction profiles of *Elp3*^*−/−*^ cells.

Not surprisingly, PRR-stimulated protein expression was found to be ELP3-dependent, with *Elp3*^*−/−*^ cells showing a profoundly altered proteome compared to WT cells. However, our study provides several specific insights regarding the requirement of innate immune responses for Elongator. We could show that, surprisingly, only specific pathways and signalling proteins were dependent on ELP3, while others were unaffected. TLR4-and RLR-stimulated IRF3-dependent gene induction required ELP3, whereas DNA- and cGAMP-dependent gene induction was largely ELP3-independent. Further, although the proteomic analysis showed reduced expression of NFκB family members, NFκB activation after LPS stimulated appeared normal. Gene induction of the NFκB -dependent cytokines *Il6* and *Tnf* was mildly affected in BMDMs lacking ELP3, but this did not translate into a reduction of IL-6 nor TNF secretion (date not shown), unlike the case for IFNβ. This is consistent with what was observed in human THP-1 cells where ELP3 shRNA significantly reduced *IFNB* but not *TNF* induction.

Why DNA sensing in macrophages avoids the need for Elongator compared to LPS and RNA sensing is currently unclear, but one likely possibility it that some other Elongator-dependent protein regulates TLR- and RLR-stimulated IRF3 which is not required for DNA-stimulated IRF3. Numerous interacting proteins regulate IRF3 activation, *via* a variety of mechanisms (27). Thus, it also possible that ELP3 deficiency leads to a repression on the expression of a protein, which is able to interact with IRF3 and prevent its activation, *via* a multitude of possible mechanisms. When cells were primed with cGAMP prior to hpRNA stimulation, the resulting gene induction was still ELP3-dependent (data not shown), suggesting that Elongator activity is still a bottleneck for a full anti-viral gene induction response even when multiple PRR pathways are active.

For TLR4, there was a two-step requirement for ELP3 in TLR-mediated type I IFN responses: firstly in type I IFN gene induction *via* IRF3 activation, and secondly in type I IFN signalling to ISGs *via* STAT1 activation. This observation led to the discovery of a general role for ELP3 in type I IFN signalling, since direct activation of cells by IFNβ treatment was also inhibited in the absence of ELP3. The restrictive influence on type I IFN signaling enforced by the absence of ELP3 likely occurred upstream of STAT1 activation, even though STAT1 protein levels were reduced in *Elp3*^*−/−*^ cells. This is because another signaling pathways dependent on STAT1, namely IFNγ signalling, was unimpaired in *Elp3*^*−/−*^ cells, suggesting that the remaining STAT1 protein expression was not rate-limiting for signalling and responses in cells lacking ELP3. IFNγ and type I IFN signaling share many components, but the tyrosine kinase TYK2 is required for type I IFN and not IFNγ signaling. In addition to type I IFN signalling, other TYK2-mediated signalling pathways, IL-4 and IL-10, were abrogated in *Elp3*^*−/−*^ cells. This data suggests that ELP3 is required for TYK2 activation in murine macrophages. Interestingly, impaired phosphorylation of TKY2 after IL-4 stimulation of cells lacking ELP3 has recently been observed (16), further supporting the hypothesis that ELP3 is required for TYK2 activation. The mechanism by which TYK2 activation is ELP3-dependent is unclear. Potentially, ELP3 may regulate the translation of an unknown protein which is required for type I IFN-mediated TYK2 activation.

Expression of many ISG proteins were found to be ELP3-dependent, but likely for a variety of reasons. In terms of explaining basal expression patterns in ELP3-deficient cells prior to IFN stimulation, some ISGs whose expression was reduced did display a very high percentage of EDCs, suggesting they could be directly regulated by elongator, but other ISGs with a high percentage of EDCs were not affected. After stimulation, increased protein expression of many ISGs was impaired, likely due to reduced type I IFN signaling capacity as discussed above. In some cases, impaired type I IFN signaling and/or absence of detectable IRF9 protein in *Elp3*^*−/−*^ cells may account for reduced basal expression given the role of tonic IFN signaling and IRF9 in basal expression of some ISGs (28).

The requirement for ELP3 for PRR responses to RNA and a TLR ligand translated to impaired host gene induction responses to RNA viruses, namely IAV and RSV. Thus, individuals with mutations in ELP subunits may be expected to display increased susceptibility to RNA virus infections in particular, although most of the focus to date on human elongator mutations relates to neurological disorders (29). However, mouse models with ELP3 deficiency in specific immune cells do show dysregulated immune responses *in vivo* (16, 30).

In conclusion, our study shows that ELP3 and thus Elongator is required for macrophages to increase IFN signaling after PRR stimulation in order to respond to viruses effectively. These data reveal specific roles for Elongator in PRR signaling and illustrate the underappreciated importance of translational regulation in optimal anti-pathogen innate immune responses.

## Experimental procedures

### Generation and maintenance of ELP3-deficient macrophages

Immortalised bone marrow–derived macrophages (iBMDM) generated from B6 mice expressing Cas9–GFP (no. 024858; The Jackson Laboratory), using J2 transforming retroviruses expressing Raf and Myc (31), were a gift from Kate Fitzgerald (University of Massachusetts Chan Medical School) (32). *Elp3*^*−/−*^ iBMDMs were generated with the use of sgRNA targeting the *Elp3* gene (GAGGAAAGGTGCAAAATACGT), while the control cells were generated using sgRNA targeting GFP (AGCTGGACGGCGACGTAAA). sgRNAs were delivered to Cas9-GFP expressing iBMDMs *via* electroporation with the use of Neon Transfection System (Thermo Fisher Scientfic). Single cell clones were then screened *via* immunoblotting to identify the successful knock out cells. Cells were grown in DMEM with GlutaMAX medium supplemented with 10% (v/v) FCS and 50 μg/ml penicillin/streptomycin. Cells were cultured at 37 ^o^C and 5% CO_2_ and sub-cultured upon reaching 80 to 85% confluency.

### ShRNA knockdown in THP-1 cells

THP-1 cells, purchased from the European Collection of Authenticated Cell Cultures, were grown in RPMI medium supplemented with 10% (v/v) FCS and 50 μg/ml penicillin/streptomycin and cultured in suspension at 37 ^o^C and 5% CO_2_. shRNA knockdown cells were generated using a lentiviral delivery system utilizing pLKO.1-puro (Addgene #8453) shRNA expression vector, pMD2.G (Addgene #12259) lentiviral envelope and psPAX2 (Addgene #12260) lentiviral packaging vector. ShRNA targeting human *ELP3* mRNA (5′- TTACTGCATGAAGCGACATTT-3′) and negative control shRNA (5′-CAACAAGATGAAGAGCACCAA-3′) were cloned into pLKO.1 puro vector using AgeI (NEB #R3552) and EcoRI (NEB #R3101) restriction enzymes following the recommended protocol (https://www.addgene.org/protocols/plko/). The combination of lentiviral vectors was then transfected into HEK293 T cells using liposomes (Lipofectamine 2000; Invitrogen #11668019) and supernatants containing lentiviruses were harvested 48 and 72 h post transfection. THP-1 cells were then infected with control or *ELP3* shRNA lentiviruses by spinoculating 1 x 10^6^ cells at 800g for 40 min with addition of virus containing supernatant and 8 μg/ml of Polybrene (Merck #H9268). Infected THP-1s were selected with 5 μg/ml of puromycin (Merck #P8833) 48 to 72 h post viral transduction. Cells that survived the selection were then maintained in complete RPMI medium supplemented with 1 μg/ml of Puromycin and *ELP3* knock down was evaluated by qPCR using specific primers (Table 1). For the experiments, control and *ELP3* shRNA THP-1 cells were seeded at 5 x 10^5^ cells/ml in complete RPMI medium with addition of 60 ng/ml of PMA and differentiated for 24 h prior to stimulation.

**Table 1:** qPCR primers used in the study

Gene	Forward (5′- 3′)	Reverse (5′- 3′)
*Actin*	TCCAGCCTTCCTTCTTGGGT	GCACTGTGTTGGCATAGAGGT
*Ifnb*	ATGGTGGTCCGAGCAGAGAT	CCACCACTCATTCTGAGGCA
*Ifna*	ACCCTCCTAGACTCATTCTG	GTTTCTTCTCTCTCAGGTACAC
*Irf7*	TTGGATCTACTGTGGGCCCA	CTTGCCAGAAATGATCCTGGG
*Irf3*	TGAGTTTGTGACTCCAGGGG	GTAGGTTTTCCTGGGAGTGAG
*Irf2*	CTGGAGGAGCAGATAAATTCC	GTATGGATCGCCCAGTTTC
*Irf5*	GGCTTCAGTGGGTCAAC	GTGTACTTCCCTGTCTCTTTAG
*Cxcl10*	TCTGAGTGGGACTCAAGGGAT	TCGTGGCAATGATCTCAACACG
*Ccl5*	CTCACCATATGGCTCGGACA	ACAAACACGACTGCAAGATTGG
*Stat1*	TCACAGTGGTTCGAGCTTCAG	GCAAACGAGACATCATAGGCA
*Stat3*	GGGCATTTTTATGGCTTTCAAT	GTTAACCCAGGCACACAGACTTC
*Arg1*	CTCCAAGCCAAAGTCCTTAGA	AGGAGCTGTCATTAGGGACATC
*Socs3*	GCAGGAGAGCGGATTCTACT	ACGCTCAACGTGAAGAAGTG
*Irf1*	CCATTCACACAGGCCGATAC	GCCCTTGTTCCTACTCTGATC
*Isg15*	CTAGAGCCTGCAGCAATG	CACCAATCTTCTGGGCAATC
*HuELP3*	CCAAACGTGGGACTAGAAAG	ATCACCAGGGTAGGATAGAG
*HuACTIN*	CCGCGAGAAGATGACCCAGATC	GCCAGAGGCGTACAGGGATA
*HuIFNB*	CTTTGCTCTGGCACAACAGG	GTGGAGAAGCACAACAGGAGA
*HuTNF*	GCCCATGTTGTAGCAAACCC	TATCTCTCAGCTCCACGCCA
*HuCXCL10*	GGCATTCAAGGAGTACCTCTCT	GCAATGATCTCAACACGTGGAC

Primers are for mouse genes unless otherwise stated as *Hu* for human.

### Macrophage stimulation

Cells were stimulated with 100 ng/ml LPS (E.coli serotype EH100 (Ra) from Enzo), 1000 U/ml IFNβ, 50 ng/ml IFNγ, 10 ng/ml IL-10, 10 ng/ml IL-4, 10 μg/ml poly(I:C), 200 ng/ml 3p-hpRNA (Invivogen), 2.5 μg/ml VACV-70mer (33), 5 μg/ml 2′-3′cGAMP (Invivogen), 5 MOI of influenza A virus (IAV, A/Puerto Rico/8/34 (PR8): mouse adapted H1N1 IAV strain, a gift from B van den Hoogen (Erasmus Medical Center)), 5 MOI of respiratory syncytial virus (RSV, A2 strain, a gift from Bernadette van den Hoogen (Erasmus Medical Center)).

3p-hpRNA, poly(I:C), VACV-70mer and cGAMP were transfected into cells using lipofectamine 2000. For transfection, lipofectamine was used at a concentration of 1 μg/ml. Both lipofectamine and the nucleic acid were diluted to the appropriate concentration in Opti-MEM medium and allowed to stand for 5 min in microcentrifuge tubes. The two tubes were then mixed to give a homogenous solution, and incubated for 20 min at room temperature before addition to cells seeded in cell culture plates.

For virus inoculation, the medium was removed from adhered BMDMs in cell culture plates, and cells were washed using 1 X PBS. An inoculum composed of either IAV or RSV and media was then added to cells and plates were placed in incubator at 37 °C and 5% CO_2_ for 1 h for IAV or 2 h for RSV. The inoculum was then removed and cells washed with 1 X PBS, and DMEM added to the wells. Cells were incubated for the times indicated in figure legends at 37 °C and 5% CO_2_.

### tRNA modification analysis

Total RNA was extracted from WT and *Elp3*^*−/−*^ BMDMs and sent to Arraystar Inc for tRNA modification analysis, which profiles 53 nucleoside modifications on tRNA. tRNA was isolated from total RNA by Urea-PAGE electrophoresis, then hydrolyzed and dephosphorylated to prepare single nucleosides. Nucleosides were analyzed by LC-MS on an Agilent mass spectrometer. LC-MS data was acquired using Agilent Qualitative Analysis software, and multiple reaction monitoring peaks of each modified nucleoside extracted and normalized to unmodified nucleosides.

### MTT cell viability assay

MTT was used to measure cell metabolic activity, as an indicator of cell viability. Cells were seeded at 5 × 10^5^ cells/ml in 96 well plates, allowed to adhere overnight, and stimulated as indicated the next day. Supernatants were carefully aspirated off. 200 μl of 1 mg/ml MTT was added to each well and cells were incubated at 37 °C for 2 h. MTT reagent was carefully removed using a pipette and 200 μl DMSO was added to each well to solubilize the crystals. The plate was incubated at 37 °C for 30 min and absorbance was read at 595 nm on a SpectraMax ABS (Molecular Devices) Microplate Reader using the SoftMax. Pro 7.1 software (www.moleculardevices.com).

### Cell culture and sample preparation for quantitative proteomics

For sample preparation, WT and *Elp3*^*−/−*^ iBMDMs were seeded in quadruplicate in 6-well plates at 5 × 10^5^ cells/ml (Fig. 2*A*). Cells were stimulated with LPS (100 ng/ml) for 6 or 12 h. Cells were harvested on ice, centrifuged at 180 g for 5 min, and washed with ice-cold 1 X PBS. Cell suspensions were centrifuged again before being snap-frozen in liquid nitrogen. To generate peptides for MS analysis, pellets were thawed on ice and lysed in 200 μl SDS lysis buffer (4% SDS, 10 mM DTT, 55 mM IAA, 50 mM Tris/HCl pH 7.5), then boiled at 95 °C for 5 min. Samples were sonicated at 4 °C for 20 min using a Bioruptor until a homogeneous suspension was achieved. Protein concentration was determined using the DC Protein Assay (BioRad) and adjusted to 1 μg/μl with SDS lysis buffer. For each sample, 200 μg of protein was precipitated with pre-chilled 80% (v/v) acetone at −20 °C and incubated for 3 h at −20 °C. The resulting precipitates were pelleted, washed once with 80% acetone, air dried at room temperature, and resolubilized in 80 μl thiourea buffer (6 M urea, 2 M thiourea in 10 mM Hepes, pH 8.0), followed by sonication for 10 min at 4 °C. For protein digestion, 25% of the resolubilized sample was diluted 1:5 with Trypsin-Protease-Mix (100 mM Tris/HCl pH 8, 1 μg trypsin; Promega) and incubated for 12 h at room temperature. TFA and acetonitrile were then added to final concentrations of 0.6% and 2%, respectively. Peptides were desalted and concentrated using C18 Empore filter discs (3M).

### Liquid Chromatography–Tandem mass spectrometry

Peptides were loaded on a 50 cm reverse-phase analytical column (75 μm diameter, 60 °C; ReproSil-Pur C18-AQ 1.9 μm resin; Dr Maisch), separated using an EASY-nLC 1200 system and directly analyzed on a Q-Exactive HF mass spectrometer equipped with a nano-electrospray source (all of them from Thermo Fisher Scientific). The mass spectrometer was operated in positive ionization mode, the spray voltage was set to 2.4 kV, funnel RF level at 60, and heated capillary at 250 °C. Peptides were separated using a 120 min gradient at a flow rate of 300 nl/min, and a binary buffer system consisting of buffer A 0.1% (v/v) formic acid in water, and buffer B 80% (v/v) acetonitrile, 0.1% (v/v) formic acid in water. More in detail: 5 to 30% (95 min), 30 to 95% (10 min), wash out at 95% for 5 min, readjustment to 5% in 5 min, and kept at 5% for 5 min. Data-dependent acquisition included repeating cycles of one MS1 full scan (300–1 650 m/z, R = 60,000 at 200 m/z) at an ion target of 3 × 10^6^ with injection time of 20 ms. For MS2 scans the top 15 intense isolated and fragmented peptide precursors (R = 15,000 at 200 m/z, ion target value of 1 × 10^5^, and maximum injection time of 25 ms) were recorded. Dynamic exclusion, isolation window of the quadrupole, and HCD normalized collision energy were set to 20 s, 1.4 m/z, and 27%, respectively.

### Protein identification

Protein group identification was performed in MaxQuant version 2.6.6.0 (maxquant.org), with a false discovery rate of 0.01, label free quantification (LFQ) and match between runs. All other parameters were set to default. The reference proteome used was *Mus musculus* (UniProtKB proteome UP000000589, version revisited on 25th February 2025). Subsequent filtering and data processing were carried out in Perseus (version 2.3.3.0). The following filters were applied: contaminants, proteins identified only by site and reverse matches. LFQ values were log-transformed and retained for further analysis if at least 70% of LFQ values were present within each group. Missing values were imputed using a Gaussian distribution with a width of 0.3 and a downshift of 1.8, according to the default parameters of Perseus. Functional annotation to GO Biological Process terms was performed in Perseus. Volcano plots were generated using an in-house custom python script, utilizing the libraries pandas, matplotlib.pyplot, seaborn, numpy, scipy.stats, statsmodels.stats.multitest, re, and adjustText. Q-values were calculated using the scipy.stats module. Bar plots were generated using LFQ values that were not filtered for NaN values and not imputed. Missing values were treated as non-detectable (*i*.*e*., set to zero). Statistical analysis was performed using the Mann-Whitney U test to assess differences between groups.

### Gene ontology biological process enrichment analysis

GO enrichment analysis of biological processes was performed on significantly upregulated or downregulated proteins identified in each comparison: ELP3^−/−^ *versus* WT at 0 h (mock), 6 h LPS stimulation, and 12 h LPS stimulation. The reference background included all proteins detected across the entire proteomics dataset.

Enrichment of GO Biological Process Direct terms was carried out using the DAVID Functional Annotation Tool (https://davidbioinformatics.nih.gov/tools.jsp). A minimum protein count of 3 and a Benjamini–Hochberg adjusted *p*-value cutoff of 0.1 were applied.

Significant GO terms were visualized as heatmaps generated in Python using the matplotlib.pyplot and seaborn libraries. No terms met the significance threshold in the upregulated protein sets; therefore, only results from the downregulated sets are shown.

### Immunoblotting

Table 2 shows antibodies used for immunoblotting, source and dilution used. All antibodies are commercially available and have been validated by the manufacturers and used by other publications. Cells were treated as described in figure legends and then harvested post stimulation on ice. Supernatants were removed and cells were washed with 1 X ice-cold PBS. Sample buffer containing DTT was added into the wells and cells were scraped and transferred to microcentrifuge tubes. Lysates were boiled for 5 min at 95 °C. Lysates were then subjected SDS-PAGE. Proteins were transferred from gels to nitrocellulose membrane by semi-dry transfer and membranes blocked in 5% (w/v) BSA in 0.1% (v/v) TBS-Tween for 1 h at room temperature. Membranes were then incubated with primary antibodies overnight at 4 °C at the indicated dilutions (Table 2). Following primary antibody incubation, membranes were washed 4 times in 0.1% (v/v) TBS-Tween. Washed membranes were incubated with secondary antibody for 1 h at room temperature, and then washed 4 more times prior to imaging using the Odyssey Imaging System (LI-COR Biosciences).

**Table 2:** Antibodies used for immunoblotting

Antibody	Raised in	Company	Cat. #	Dilution
β-Actin	Mouse	Sigma-Aldrich	A5316	1:2000
ELP3	Rabbit	Abcam	ab190907	1:2000
IRF3	Rabbit	Santa cruz	sc-9082	1:1000
phospho-IRF3	Rabbit	Cell signalling technology (CST)	4947	1:500
STAT1	Rabbit	CST	9172	1:1000
phospho-STAT1	Rabbit	CST	9167	1:1000
STAT2	Mouse	CST	sc-514193	1:1000
phospho-STAT2	Rabbit	Millipore	07–224	1:1000
p65	Mouse	Santa cruz	sc-8008	1:1000
phospho-P65	Rabbit	CST	3033S	1:1000
TYK2	Rabbit	Proteintech	16412-1-AP	1:1000
JAK1	Rabbit	CST	3332S	1:1000
IFNAR1	Rabbit	Thermo Fisher	PA5-79442	1:1000
Mouse IRDye 680LT secondary	Goat	LiCOR	926–68070	1:10,000
Rabbit IRDye 800CW secondary	Goat	LiCOR	926–32211	1:10,000

### RNA analysis by quantitative RT-PCR

Cells were stimulated for times indicated in figure legends, and RNA was extracted from cells in culture using the High Pure RNA isolation kit (Roche) according to the manufacturer’s instructions. Reverse transcription with random hexamers was performed using Moloney murine leukemia virus reverse transcriptase (Promega) following the manufacturer’s protocol. mRNA was quantified with PowerUp SYBR green (Applied Biosystems) using primer pairs targeting genes of interest (see Table 1). RT-PCR was performed using QuantiStudio 3 (Applied Biosystems) Real-Time PCR system (Invitrogen). Relative mRNA expression was calculated using the comparative C_T_ method, normalising the gene of interest to the housekeeping gene *β-actin*.

### ELISA for IFNβ secretion

ELISA plates were coated with a monoclonal rat anti-mouse IFNβ capture antibody (Santa Cruz, Cat#SC-57201) in carbonate buffer overnight at 4 °C. Plates were then blocked in blocking solution (10% FCS/PBS) at room temperature for 2 h and then incubated with sample supernatants diluted in blocking buffer for overnight at 4 °C. A polyclonal rabbit anti-mouse IFNβ (PBL InterferonSource, Cat#32400–1) in blocking solution (10% FCS/PBS) was used as a detection antibody. HRP-conjugated anti-rabbit IgG antibody (Sigma, Cat# RABHRP1) was then used for TMB substrate (BD) development. Absorbances at 450 and 620 nm wavelengths were obtained using the Spectramax ELISA plate reader (Molecular Devices) and concentrations were calculated based on a standard curve of recombinant murine IFN-β (PBL Cat#12400–1).

### Statistical analysis

Statistical analysis of mass spectrometry data is described above. For all other data, GraphPad Prism 10 (www.graphpad.com) was used to analyze data. Data are presented as mean ± SD of three independent experiments. Details of specific statistical tests used are described in figure legends and *p* values of <0.05 were considered statistically significant.

## Data availability

The mass spectrometry proteomics data have been deposited to the ProteomeXchange Consortium *via* the PRIDE partner repository with the dataset identifier PXD064630. All other data is contained in the manuscript.

## Supporting information

This article contains supporting information.

## Conflict of interest

The authors declare that they have no conflicts of interest with the contents of this article.

## References

[1] Dvorkin S., Cambier S., Volkman H.E., Stetson D.B. (2024). New frontiers in the cGAS-STING intracellular DNA-sensing pathway. Immunity.

[2] Yoneyama M., Kato H., Fujita T. (2024). Physiological functions of RIG-I-like receptors. Immunity.

[3] Kawai T., Ikegawa M., Ori D., Akira S. (2024). Decoding toll-like receptors: recent insights and perspectives in innate immunity. Immunity.

[4] Carty M., Guy C., Bowie A.G. (2021). Detection of viral infections by innate immunity. Biochem. Pharmacol..

[5] Kurata S., Weixlbaumer A., Ohtsuki T., Shimazaki T., Wada T., Kirino Y. (2008). Modified uridines with C5-methylene substituents at the first position of the tRNA anticodon stabilize U.G wobble pairing during decoding. J. Biol. Chem..

[6] Schaffrath R., Leidel S.A. (2017). Wobble uridine modifications-a reason to live, a reason to die?. RNA Biol..

[7] Suzuki T. (2021). The expanding world of tRNA modifications and their disease relevance. Nat. Rev. Mol. Cell. Biol..

[8] Hawer H., Hammermeister A., Ravichandran K.E., Glatt S., Schaffrath R., Klassen R. (2018). Roles of elongator dependent tRNA modification pathways in neurodegeneration and cancer. Genes (Basel).

[9] Lin T.Y., Abbassi N.E.H., Zakrzewski K., Chramiec-Glabik A., Jemiola-Rzeminska M., Rozycki J. (2019). The elongator subunit Elp3 is a non-canonical tRNA acetyltransferase. Nat. Commun..

[10] Chen Y.T., Hims M.M., Shetty R.S., Mull J., Liu L., Leyne M. (2009). Loss of mouse Ikbkap, a subunit of elongator, leads to transcriptional deficits and embryonic lethality that can be rescued by human IKBKAP. Mol. Cell. Biol..

[11] Cuajungco M.P., Leyne M., Mull J., Gill S.P., Lu W., Zagzag D. (2003). Tissue-specific reduction in splicing efficiency of IKBKAP due to the major mutation associated with familial dysautonomia. Am. J. Hum. Genet..

[12] Simpson C.L., Lemmens R., Miskiewicz K., Broom W.J., Hansen V.K., van Vught P.W. (2009). Variants of the elongator protein 3 (ELP3) gene are associated with motor neuron degeneration. Hum. Mol. Genet..

[13] Endres L., Fasullo M., Rose R. (2019). tRNA modification and cancer: potential for therapeutic prevention and intervention. Future Med. Chem..

[14] Huang B., Johansson M.J., Bystrom A.S. (2005). An early step in wobble uridine tRNA modification requires the Elongator complex. RNA.

[15] Goffena J., Lefcort F., Zhang Y., Lehrmann E., Chaverra M., Felig J. (2018). Elongator and codon bias regulate protein levels in mammalian peripheral neurons. Nat. Commun..

[16] Chen D., Nemazanyy I., Peulen O., Shostak K., Xu X., Tang S.C. (2022). Elp3-mediated codon-dependent translation promotes mTORC2 activation and regulates macrophage polarization. EMBO J..

[17] Mostafavi S., Yoshida H., Moodley D., LeBoite H., Rothamel K., Raj T. (2016). Parsing the interferon transcriptional network and its disease associations. Cell.

[18] Nedialkova D.D., Leidel S.A. (2015). Optimization of codon translation rates via tRNA modifications maintains proteome integrity. Cell.

[19] Zinshteyn B., Gilbert W.V. (2013). Loss of a conserved tRNA anticodon modification perturbs cellular signaling. PLoS Genet..

[20] Rapino F., Zhou Z., Roncero Sanchez A.M., Joiret M., Seca C., El Hachem N. (2021). Wobble tRNA modification and hydrophilic amino acid patterns dictate protein fate. Nat. Commun..

[21] Rapino F., Delaunay S., Rambow F., Zhou Z., Tharun L., De Tullio P. (2018). Codon-specific translation reprogramming promotes resistance to targeted therapy. Nature.

[22] Gewaid H., Bowie A.G. (2024). Regulation of type I and type III interferon induction in response to pathogen sensing. Curr. Opin. Immunol..

[23] Kurt-Jones E.A., Popova L., Kwinn L., Haynes L.M., Jones L.P., Tripp R.A. (2000). Pattern recognition receptors TLR4 and CD14 mediate response to respiratory syncytial virus. Nat. Immunol..

[24] Huang B., Lu J., Bystrom A.S. (2008). A genome-wide screen identifies genes required for formation of the wobble nucleoside 5-methoxycarbonylmethyl-2-thiouridine in Saccharomyces cerevisiae. RNA.

[25] Johansson M.J., Esberg A., Huang B., Bjork G.R., Bystrom A.S. (2008). Eukaryotic wobble uridine modifications promote a functionally redundant decoding system. Mol. Cell. Biol..

[26] Tukenmez H., Xu H., Esberg A., Bystrom A.S. (2015). The role of wobble uridine modifications in +1 translational frameshifting in eukaryotes. Nucleic. Acids. Res..

[27] Chathuranga K., Weerawardhana A., Dodantenna N., Lee J.S. (2021). Regulation of antiviral innate immune signaling and viral evasion following viral genome sensing. Exp. Mol. Med..

[28] Platanitis E., Demiroz D., Schneller A., Fischer K., Capelle C., Hartl M. (2019). A molecular switch from STAT2-IRF9 to ISGF3 underlies interferon-induced gene transcription. Nat. Commun..

[29] Gaik M., Kojic M., Wainwright B.J., Glatt S. (2023). Elongator and the role of its subcomplexes in human diseases. EMBO Mol. Med..

[30] Lemaitre P., Bai Q., Legrand C., Chariot A., Close P., Bureau F. (2021). Loss of the transfer RNA wobble uridine-modifying enzyme Elp3 delays T cell cycle entry and impairs T follicular helper cell responses through deregulation of Atf4. J. Immunol..

[31] Roberson S.M., Walker W.S. (1988). Immortalization of cloned mouse splenic macrophages with a retrovirus containing the v-raf/mil and v-myc oncogenes. Cell. Immunol..

[32] Bhatta A., Atianand M., Jiang Z., Crabtree J., Blin J., Fitzgerald K.A. (2020). A mitochondrial micropeptide is required for activation of the Nlrp3 inflammasome. J. Immunol..

[33] Unterholzner L., Keating S.E., Baran M., Horan K.A., Jensen S.B., Sharma S. (2010). IFI16 is an innate immune sensor for intracellular DNA. Nat. Immunol..

