# Supplementary material for: Elongator is required for pattern recognition receptor and type I interferon signaling in macrophages
Source: J Biol Chem. 2025 Nov 5;301(12):110916. doi: 10.1016/j.jbc.2025.110916 (PMC12702316; doi:10.1016/j.jbc.2025.110916)
Supplement: Table S1 [file mmc2.pdf]

Elongator is required for pattern recognition receptor and type I interferon signaling in macrophages.

Jamie Murphy[1], Marcin Baran[1], Corinna Grünke[2], Darya Haas[2], Gillian Barber[1], Andreas Pichlmair[2, 3, 4] and Andrew G Bowie[1*]

[1]School of Biochemistry and Immunology, Trinity Biomedical Sciences Institute, Trinity College Dublin, Dublin 2, Ireland.

[2]Institute of Virology, School of Medicine, Technical University of Munich, Munich, Germany.

[3]Institute of Virology, Helmholtz Center Munich, Munich, Germany

[4]German Centre for Infection Research (DZIF), Partner site Munich, Germany

* Correspondence: agbowie@tcd.ie

Supporting information includes Table S1 and Figures S1-S3.

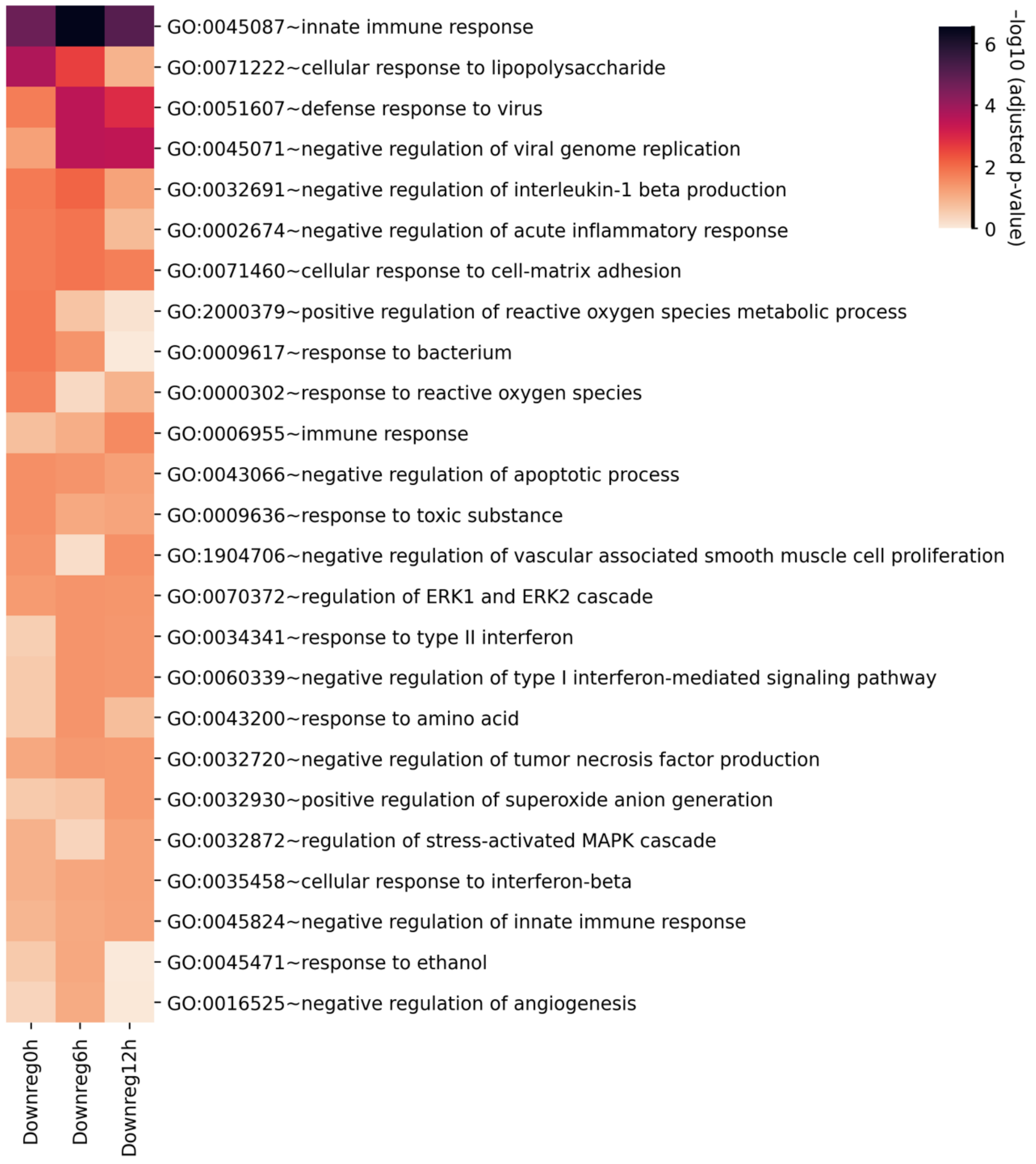

Figure S1. Gene Ontology enrichment analysis for biological processes in *Elp3*$^{-/-}$ compared to WT cells

Gene Ontology (GO) enrichment analysis of biological processes was performed on significantly upregulated or downregulated proteins identified in each comparison: *Elp3*$^{-/-}$ vs. WT at 0 h (mock), 6 h LPS stimulation, and 12 h LPS stimulation. Significant GO terms are visualized as a heatmap. No terms met the significance threshold in the upregulated protein sets; therefore, only results from the downregulated sets are shown.

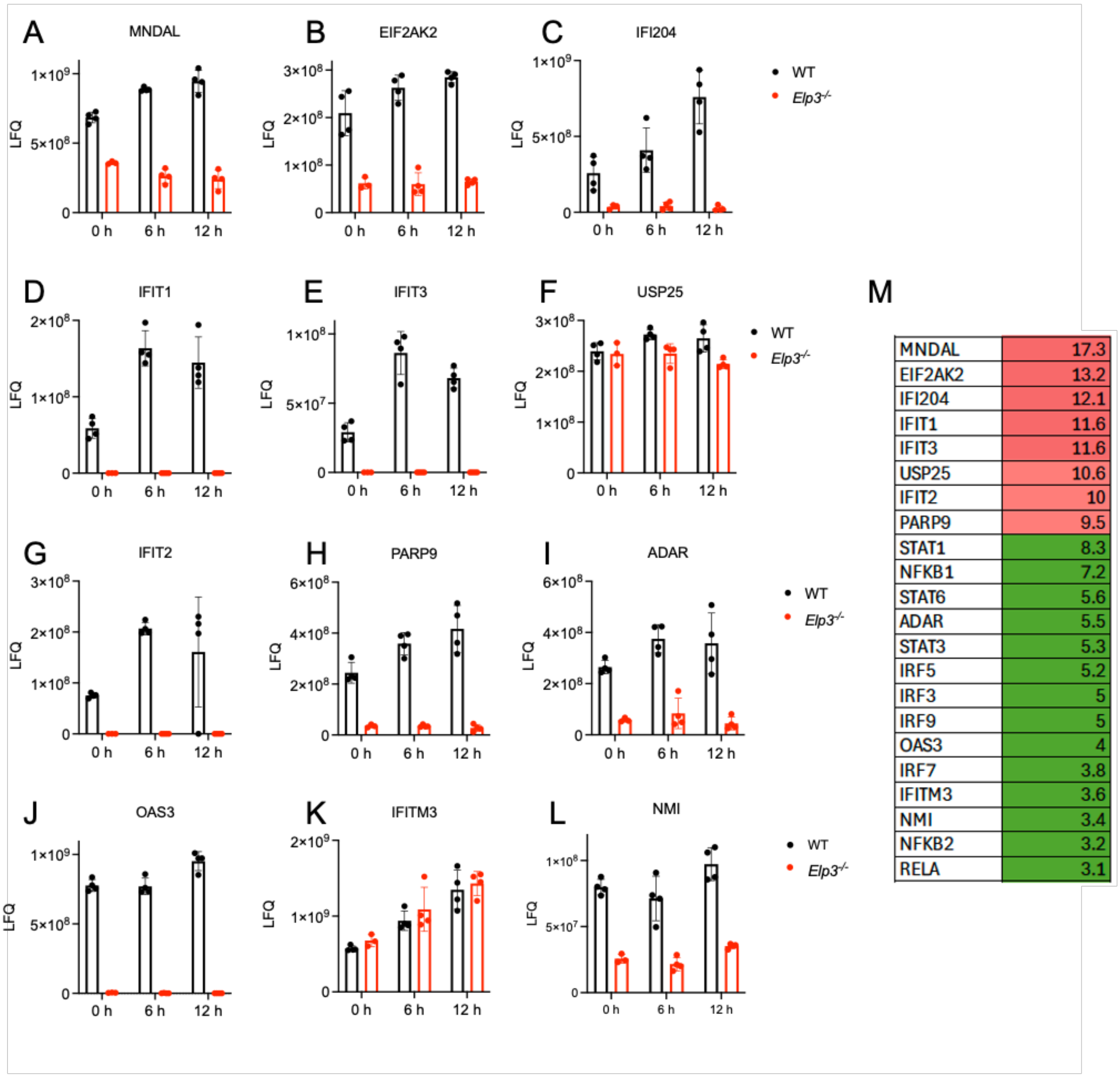

MNDAL	17.3
EIF2AK2	13.2
IFI204	12.1
IFIT1	11.6
IFIT3	11.6
USP25	10.6
IFIT2	10
PARP9	9.5
STAT1	8.3
NFKB1	7.2
STAT6	5.6
ADAR	5.5
STAT3	5.3
IRF5	5.2
IRF3	5
IRF9	5
OAS3	4
IRF7	3.8
IFITM3	3.6
NMI	3.4
NFKB2	3.2
RELA	3.1

Figure S2. Elongator dependency of ISGs.
(A-L) Label free quantification intensity values detected by mass spectrometry in WT and *Elp3*$^{-/-}$ cells stimulated for 0, 6 or 12 h with LPS for peptides from ISG-encoded proteins MNDAL (A), EIF2AK2 (B), IFI204 (C), IFIT1 (D), IFIT3 (E), USP25 (F), IFIT2 (G), PARP9 (H), ADAR (I), OAS3 (J) IFITM3 (K) and NMI (L). Data are mean ± SD for quadruplicate (or triplicate for WT 0 h sample) measurements. (M) Predicted percentage of ELP-dependent codons (EDCs) for ISGs shown here and transcription factors in Figure 4.

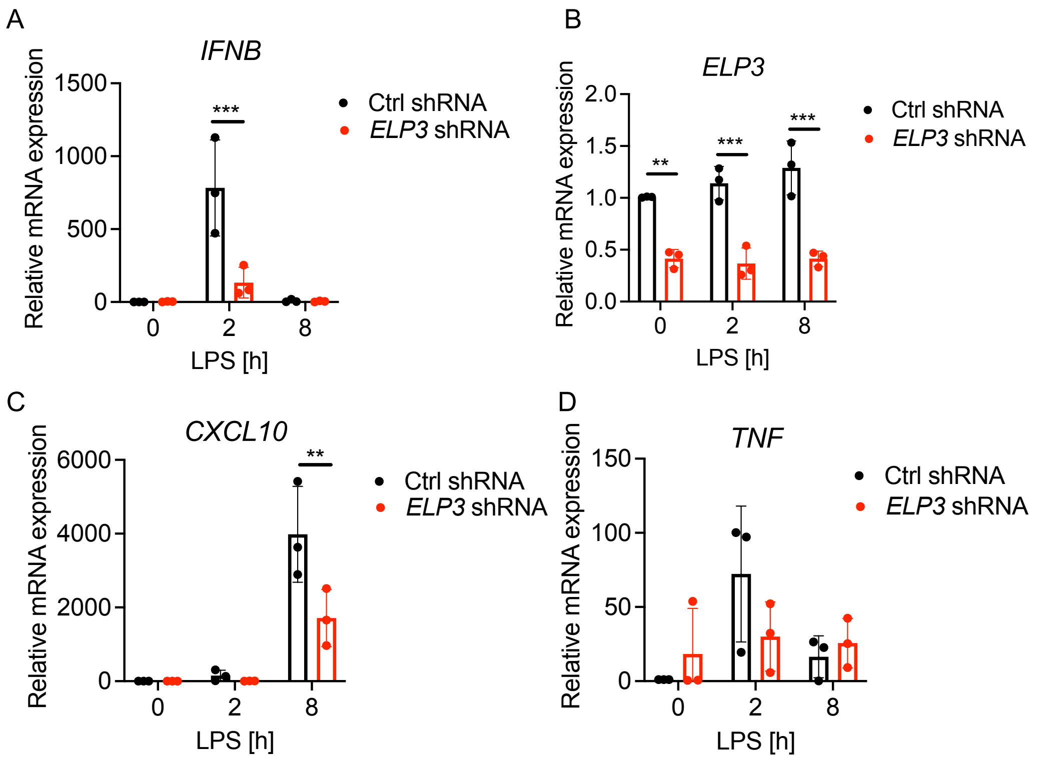

Figure S3 - ELP3 is required for LPS-stimulated IRF3-dependent genes in human THP-1 cells

(A-D) THP1 cells expressing shRNA (control or *ELP3* targeting) were stimulated with 100 ng/ml of LPS for 2 or 8 h. Following stimulation, gene expression of *ELP3* (A), *IFNB* (B), *CXCL10* (C) and *TNF* (D) was measured by real time PCR. Data are mean ± SD gene expression relative to the unstimulated control (Ctrl) shRNA sample and presented as average of 3 independent experiments, each performed in triplicate. Data significance was tested with a 2-way ANOVA using Šídák's multiple comparisons test. ** $p<0.01$ and *** $p<0.001$.
